# Exploring the Relationship of Relative Telomere Length and the Epigenetic Clock in the LipidCardio Cohort

**DOI:** 10.3390/ijms20123032

**Published:** 2019-06-21

**Authors:** Verena L. Banszerus, Valentin M. Vetter, Bastian Salewsky, Maximilian König, Ilja Demuth

**Affiliations:** 1 Charité–Universitätsmedizin Berlin, corporate member of Freie Universität Berlin, Humboldt-Universität zu Berlin and the Berlin Institute of Health, Lipid Clinic at the Interdisciplinary Metabolism Center, 13353 Berlin, Germany; verena-laura.banszerus@charite.de (V.L.B.); valentin.vetter@charite.de (V.M.V.); bastian.salewsky@gmx.de (B.S.); 2 Medizinische Klinik mit Schwerpunkt Nephrologie und Intensivmedizin, Charité-Universitätsmedizin Berlin, 13353 Berlin, Germany; koenig.maximilian@charite.de; 3 Berlin-Brandenburg Center for Regenerative Medicine (BCRT), Charité Universitätsmedizin Berlin, 13353 Berlin, Germany

**Keywords:** biomarker of ageing, biological age, aging, telomere length, epigenetic clock, DNA methylation (DNAm) age, DNAm age acceleration, LipidCardio Study, Berlin Aging Study II (BASE-II)

## Abstract

Telomere length has been accepted widely as a biomarker of aging. Recently, a novel candidate biomarker has been suggested to predict an individual’s chronological age with high accuracy: The epigenetic clock is based on the weighted DNA methylation (DNAm) fraction of a number of cytosine-phosphate-guanine sites (CpGs) selected by penalized regression analysis. Here, an established methylation-sensitive single nucleotide primer extension method was adapted, to estimate the epigenetic age of the 1005 participants of the LipidCardio Study, a patient cohort characterised by high prevalence of cardiovascular disease, based on a seven CpGs epigenetic clock. Furthermore, we measured relative leukocyte telomere length (rLTL) to assess the relationship between the established and the promising new measure of biological age. Both rLTL (0.79 ± 0.14) and DNAm age (69.67 ± 7.27 years) were available for 773 subjects (31.6% female; mean chronological age= 69.68 ± 11.01 years; mean DNAm age acceleration = −0.01 ± 7.83 years). While we detected a significant correlation between chronological age and DNAm age (*n* = 779, *R* = 0.69), we found neither evidence of an association between rLTL and the DNAm age (*β* = 3.00, *p* = 0.18) nor rLTL and the DNAm age acceleration (*β* = 2.76, *p* = 0.22) in the studied cohort, suggesting that DNAm age and rLTL measure different aspects of biological age.

## 1. Introduction

Aging is accompanied by a range of DNA modifications. Telomere length, which shortens as a consequence of DNA replication, has been widely accepted as a biomarker of aging [1]. While being inversely correlated with chronological age [2,3], telomere length is also associated with a range of age-associated phenotypes and clinical diseases [4,5]. These include cardiovascular disease and its risk factors: smoking and alcohol abuse, hypertension, obesity, physical inactivity, metabolic syndrome, diabetes mellitus type II, and atherosclerosis [6,7,8,9,10,11].

Recently, a novel candidate epigenetic biomarker of aging has been shown to predict an individual’s chronological age with high accuracy: The epigenetic clock is based on the weighted DNA methylation fraction of a number of cytosine-phosphate-guanine sites (CpGs) selected by an elastic net (or lasso) penalized regression analysis with chronological age as the dependent variable [12,13]. Since Horvath and Hannum et al. characterized their CpGs-subsets for epigenetic age estimation in 2013 (including 353 and 71 CpGs, respectively), further epigenetic clocks have been described by others [12,13,14,15]. Different clocks were established as a consequence of the training set population studied and the choice and number of CpGs included, which is linked with the level of accuracy of chronological age prediction.

Interestingly, DNA methylation (DNAm) age correlates with cell passage number in vitro and can be predicted across different tissues, including non-proliferating ones in vivo, suggesting that DNA methylation is not exclusively reflecting mitotic age [12]. This is in line with the finding that DNAm age and relative leucocyte telomere length (rLTL) were independently associated with chronological age and mortality [16,17]. The few existing studies found no supporting evidence of a significant association between rLTL and DNAm age [18,19,20] or reported a weak association [16]. Moreover, rLTL was reported to have a lower predictive power in estimating chronological age in comparison to the epigenetic clock [16,21].

While the number of studies reporting a positive correlation between DNAm and chronological age in a range of different study populations rises, there is accumulating evidence suggesting that DNAm age somewhat reflects biological age [19,21,22,23]. Moreover, DNA methylation age was reported to correlate with a range of investigated aging-related phenotypes and clinical diseases [17,19,22,24,25]. This includes cardiovascular disease [26]. Under the assumption that DNA methylation age reflects biological age, calculating the deviation of the epigenetic age estimate and the chronological age gives rise to a second potentially clinically relevant measure: DNAm age acceleration. The term encompasses both the positive and the negative discrepancy between biological age estimated by an epigenetic clock of choice and the chronological age, indicating an acceleration or deceleration of the individual’s rate of aging respectively and in comparison with the study cohort. Here we aim to explore the association of rLTL and the epigenetic clock variables, DNAm age and DNAm age acceleration, in the context of cardiovascular disease in the LipidCardio cohort.

## 2. Results

**Figure A1:**
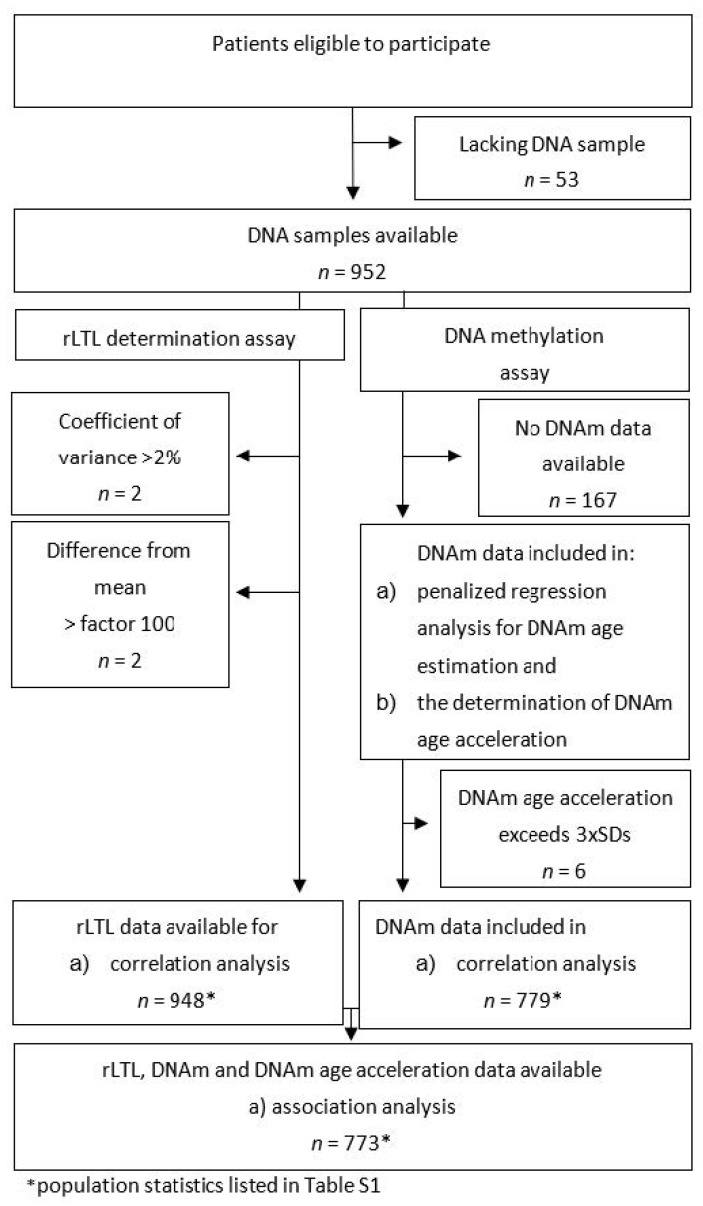
Composition and sample size (*n*) of the LipidCardio study (sub)populations.

The cohort’s mean chronological age was 69.68 ± 11.01 years (*n* = 773), ranging from 21.28 to 91.22 years (Table 1). 31.6 % of the participants are female and 97.7 % are of Caucasian ancestry. The cohort is characterized by a high prevalence of cardiovascular risk factors, including an increased mean bodyweight, combined with low physical activity (data shown elsewhere [27]) and diabetes mellitus type II affecting 26.9% of the subjects. 67.2% of the participants reported to be active smokers or ex-smokers, adding to a mean of 30.15 ± 28.9 pack years (*n* = 463), while 56.0% reported to consume alcohol, with a mean intake of 5.2 ± 6.2 units weakly (1 unit = 0.33L beer = 0.25L wine = 0.02L liquor). 80.7% of the subjects were affected from hypertension, 75.8% had coronary heart disease, and 30.4% reported a history of myocardial infarction. 953 genomic DNA samples (from 95% of the LipidCardio participants) were available to undergo laboratory analysis for rLTL determination and the DNA methylation assay (Table S1). Detailed information on all subgroups studied (arising from dealing with missing values by pairwise deletion) can be found in Figure A1 and Table S1. Clinical differences in the characteristics of the subgroups studied were minor. Complete data sets on rLTL and DNAm age, as well as DNAm age acceleration (estimated by the residuals of the regression of the chronological age on the DNA methylation age) were obtained for 773 (76.9%) participants (population statistics listed in Table 1).

### 2.1. Relative Leukocyte Telomere Length in LipidCardio

The determination of rLTL succeeded in 948 (99.5%) of the DNA samples. rLTL was measured in triplicates and in comparison to a reference sample mix. Two samples were excluded, since the triplicate rLTL measurements exceeded the coefficient of variance cut-off of 2% and three samples were excluded, since the individual’s rLTL differed from the cohort’s mean rLTL by more than factor of hundred (Figure A1). The analysis for rLTL yielded a mean relative length of 0.79 ± 0.14 (*n* = 948) ranging from 0.10 to 1.22. Mean rLTL was significantly shorter (*p* = 1.6 × 10^−8^) in female (mean = 0.75, *n* = 281) compared to male subjects (mean = 0.80, *n* = 667). We did not detect a correlation between rLTL and chronological age in our cohort (*n* = 948; *R_s_*^2^ = 3.59 × 10^−4^) (Figure S1).

### 2.2. DNA Methylation (DNAm) Age Estimation and DNAm Age Acceleration in LipidCardio

A complete set of data on the fraction of DNA methylation measured at eight CpGs was available for 785 (82.5%) of the DNA samples and were included in the stepwise-penalized linear regression analysis to determine the line of best fit for the estimation of the DNAm age (Figure A1). 167 did not meet the predetermined quality standards and were excluded. Separate correlation analysis showed that the methylation status at seven out of eight CpGs (except for cg24768561) were correlated significantly with chronological age in the LipidCardio cohort (Figure S2a–h).

Interestingly, the CpGs’s methylation fraction at the DNA site cg25809905, did not improve the fit of the model for DNAm determination significantly (*p* = 0.07, Table 2). Consequently, we estimated the DNAm age based on a *seven* CpGs epigenetic clock. As shown previously [14,28], the methylation status at two CpGs (cg16386080 and cg24768561) were positively associated with chronological age as determined by linear regression analysis, while the remaining were inversely associated (Table 2).

**Figure 1:**
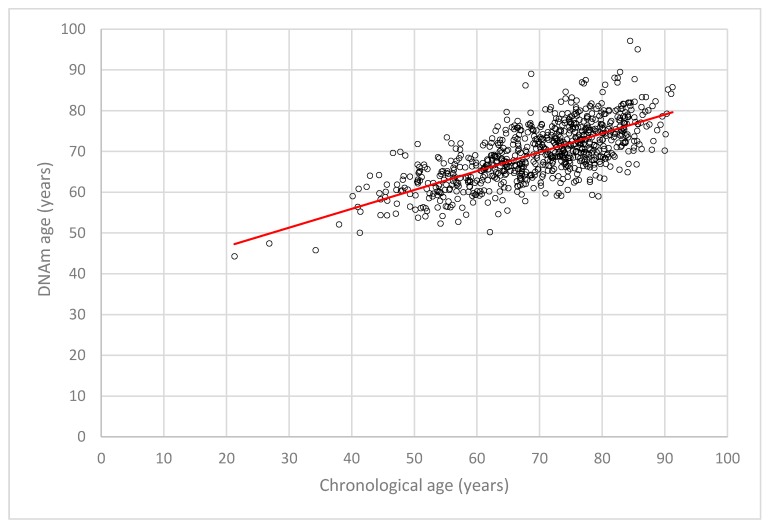
Scattered plot depicting the linear correlation of DNAm age with the chronological age (*n* = 779, *R* = 0.69).

The mean DNAm age of the LipidCardio participants was estimated 69.66 ± 7.24 years (*n* = 779, range 44.30 to 97.14 years) with a mean DNAm age acceleration of −0.01 ± 7.81 years (*n* = 779, range −19.84 to 23.52 years), while participants’ mean chronological age was 69.68 ± 11.01 years (*n* = 773, ranging from 21.28 to 91.22 years). Six samples were excluded from the above statistics, since the DNAm age acceleration exceeded three standard deviations of the mean. A correlation analysis showed that DNAm age estimated by the seven CpG epigenetic clock and the chronological age are correlated with *R* = 0.69 (*n* = 779, Figure 1) in the LipidCardio cohort. A significant association between DNAm age and chronological age prevailed after correcting for sex and lifestyle factors such as smoking and alcohol consumption (*β* = 0.503, SE = 0.31, *p* = 1.50 × 10^−40^).

A stratification of the data for sex indicated a comparable, but marginally higher power of the seven CpGs epigenetic clock to predict the chronological age of male patients compared to female patients (female: *n* = 246, *R* = 0.65; male: *n* = 533, *R* = 0.71). This was supported by a significant difference (*p* = 2.51 × 10^−3^) in mean DNAm age acceleration, which was closer to zero in male subjects (*n* = 537, 0.80 ± 8.12 years) compared to female subjects (*n* = 248, −1.74 ± 8.24 years).

### 2.3. Comparison of Different DNA Methylation (DNAm) Age Acceleration Estimates in the Berlin Aging Study II (BASE-II)

To estimate the magnitude of the effect of age-associated changes of the blood cell distribution on the DNAm age acceleration, we retrieved data on chronological age, DNAm age, and intrinsic epigenetic age acceleration (IEAA) from a cohort of predominantly healthy subjects of the Berlin Aging Study II (BASE-II, age group: ≥60 years of chronological age, *n* = 1395, mean chronological age: 68.7 ± 3.7 years, 49.3% female) [16]. IAEE was defined as the residuals from regressing the chronological age on the DNAm age, adjusting for the individual’s neutrophil, monocyte, lymphocyte, and eosinophil count. In addition to the IEAA determined by Vetter and colleagues, DNAm age acceleration was determined by two alternative methods: first, by the difference of DNAm age and chronological age, and second, by the residuals from regressing the chronological age on the DNAm age, without adjusting for the individual’s blood cell distribution. Both the difference (*n* = 1395, −2.46 ± −7.23 years, *R* = 0.96) and the residuals (−2.00 ± −7.37 years, *R* = 0.96) were highly correlated with IEAA and with each other (0.02 ± 6.91 years, *R* = 0.99) (Figure S3). While the mean difference underestimated the DNAm age acceleration marginally, IEAA overestimated the age acceleration determined by residuals slightly in the BASE-II cohort in comparison with the residuals.

Of note, in both cohorts, BASE-II and LipidCardio, DNAm fractions were analysed according to the same laboratory protocol and DNAm age was calculated based on a seven CpGs epigenetic clock, out of which six CpGs were identical [16].

### 2.4. Accuracy of Chronological Age Prediction by the Seven Cytosine-Phosphate-Guanine Sites (CpGs) Epigenetic Clock Across Age Groups

In the LipidCardio cohort, chronological age and DNAm age acceleration were negatively correlated (*n* = 773, *R*= −0.75). To explore this further and to examine the accuracy of prediction of the seven CpGs epigenetic clock across different age groups, we stratified participants into four groups according to their chronological age: subjects 60 years of age or younger, between 60 and 70 years, between 70 and 80 years, and 80 years and above. Mean chronological age, mean DNAm age acceleration, and their standard deviations were determined for each group. (Table S2).

The chronological age prediction of the employed seven CpGs epigenetic clock was most accurate for the age group of 60 to 80 years of chronological age, with the smallest difference from the cohort’s mean chronological age. The DNAm age of younger and older participants tend to be over- or underestimated respectively, which is contributed (in part) by the statistical approach employed.

### 2.5. Relative Leukocyte Telomere Length (rLTL), DNA Methylation (DNAm) Age, and DNAm Age Acceleration in the LipidCardio Cohort

To examine the relationship between the two epigenetic clock variables and rLTL, DNAm age and DNAm age acceleration were regressed separately on rLTL, stepwise adjusting for chronological age, sex, and lifestyle factors (Table 3).

Neither DNAm age (*n* = 773, *p* = 0.12), nor DNAm age acceleration (*n* = 773, *p* = 0.26) were associated with rLTL in the LipidCardio cohort (Table 3 and Figure S4a,b).

## 3. Discussion

The aim of this study was to explore the association of rLTL, an established biomarker of aging, and the novel epigenetic clock biomarkers, DNAm age and DNAm age acceleration, in a cohort with a high prevalence of cardiovascular disease. To address this aim, a total of 953 genomic DNA samples, representing about 95% of the LipidCardio sample (*n* = 1005) were analysed to determine rLTL, DNAm age, and DNAm age acceleration.

### 3.1. Relative Leukocyte Telomere Length and Chronological Age

An inverse correlation of chronological age and telomere length is well-documented within the literature. Telomere length, shortening as a consequence of DNA replication, has been accepted as a biomarker of aging with compelling molecular genetic evidence [1,2,3,29,30]. However, we did not detect an association between chronological age and rLTL in our cohort.

This may be explained by an insufficiency of the chronological age range studied (95% confidence interval between 58.87 and 80.99 years). In the BASE-II cohort, rLTL and chronological age were not significantly correlated in the subpopulation of 60- to 84-year-olds (*n* = 1407; *p* = 0.88), but rLTL was significantly shorter in the older subpopulation, compared to the younger one (chronological age: 20–37 years; *n* = 402, *p* = 1.9 × 10^−10^), supporting the above considerations [16].

Moreover, a correlation of chronological age and rLTL might be obscured by a large variability of the individuals’ rLTL in the LipidCardio cohort. High inter-individual variability in telomere length has been reported as a consequence of genetic variance, sex, and lifestyle factors, including physical activity and bodyweight, psychological stress, smoking, and alcohol consumption [30]. Shortened telomeres or an acceleration of telomere attrition have been associated with cardiovascular disease, including hypertension, coronary heart disease, risk of stroke, and myocardial infarction [30,31,32], which are prevalent in the LipidCardio cohort and may have contributed to the inter-individual variability in rLTL.

Interestingly, mean rLTL was significantly shorter in female compared to male subjects in the LipidCardio cohort (*p* = 1.6 × 10^−8^). A meta-analysis based on 36,230 subjects from 36 cohorts by Gardner el al. reported previously that female telomeres were detected to be significantly longer compared to male telomeres, in an analysis including different methods of telomere length determination [33]. However, an underlying technical bias was revealed when stratifying for the laboratory method employed [33].

In line with the above finding, telomere length was reported to be longer in male subjects, when measured by quantitative real time polymerase chain reaction (PCR)—a finding which we discussed previously in the context of similar results yielded in the Berlin Aging Study II [16,34].

Moreover, rLTL determination by qPCR lacks single cell or even single chromosome resolution. Consequently (and limiting to this study), only the average rLTL of the individual subjects were studied here [35].

### 3.2. The Seven Cytosine-Phosphate-Guanine Sites (CpGs) Epigenetic Clock

DNAm age, determined by a seven CpGs epigenetic clock and chronological were correlated with *R* = 0.69 (*n* = 779). While studies which employed the Horvath or Hannum clock (including 353 and 71 CpGs, respectively) were able to predict chronological age with an accuracy approaching *R*^2^ = 0.94 and *R*^2^ = 0.81 respectively, the predictive power was compromised by the trade-off of opting for a clock based on a reduced number of CpG sites here [12,13]. Indeed, chronological age and DNAm age determined in 390 subjects by the eight CpGs epigenetic clock by Vidal-Bralo and colleagues correlated with an *R*^2^ = 0.60 [28]. In BASE-II, chronological age and DNAm age determined by a seven CpGs epigenetic clock were significantly correlated with a *R*^2^ = 0.47, which is in comparable range of the current study.

Stratified analysis of the female and male participants indicated a marginally increased predictive power of the seven CpGs epigenetic clock to predict the chronological age of male patients compared to the female ones (female: *n* = 246, *R* = 0.65; male: *n* = 533, *R* = 0.71). This may be a consequence of an overrepresentation of male participants (68.4%) in the data set employed to fit the model for DNAm age estimation. Accordingly, the deviation of the individual’s DNAm age measured by DNAm age acceleration was significantly higher (*p* = 2.51 × 10^−3^) in female subjects (*n* = 248; −1.74 ± 8.24 years) compared to male participants (*n* = 537; 0.80 ± 8.12 years).

### 3.3. DNAm Age Acceleration

Previously different methods to quantify DNAm age have been employed: Horvath initially defined DNAm age acceleration as the difference between DNAm age and chronological age, while Hannum et al. determined an individual’s apparent methylation aging rate (AMAR) by the ratio of DNAm age estimate to chronological age [12,13].

Whole blood consists of a heterogeneous mixture of blood cells with diverging methylation profiles and fluctuating cell composition during the individual’s course of aging [36,37]. Thus, it has been suggested to adjust an individual’s DNAm age acceleration for the blood cell distribution, a measure termed cell-intrinsic epigenetic age acceleration measures or IEAA [22,38]. IEAA is distinguished from cell-extrinsic epigenetic age acceleration measures (EEAA), which is estimated independent of the individual’s (age-associated) blood cell composition [22,38]. Both of the following methods omit to adjust for the blood cell distribution: firstly, the difference of DNAm age and chronological age, and secondly, the *residuals* from regressing the chronological age on the DNAm age.

The comparison of the three different methods to determine DNAm age acceleration showed a close correlation of the DNAm age acceleration estimates yielded from the *difference*, the *residuals*, and IEAA method (*R* = 0.94–0.99) in the BASE-II cohort. Hence, we conclude that the unavailability of data on the leukocyte distribution is a negligible limitation of the LipidCardio study set up.

### 3.4. Relative Leukocyte Telomere Length (rLTL), DNA Methylation (DNAm) Age and DNAm Age Acceleration

We found neither evidence of an association between rLTL and DNAm age (*n* = 773, *R* = 0.045), nor an association between rLTL and DNAm age acceleration (*n* = 773, *R* = 0.03) in the LipidCardio cohort. The relationship between rLTL and DNAm age or DNAm age acceleration has been examined in different cohorts previously (Table S4):

Marioni and colleges accessed two Scottish cohorts (cohort LBC1936 and LBC1921), including a total of 1354 subjects longitudinally (age: 70–90 years) [17]. Telomere length and DNAm age estimated by the Hannum epigenetic clock were reported to be independently associated with chronological age in the Scottish cohorts. Belsky and colleagues, who followed up a one-year birth cohort of 1037 subjects till the age of 38 years (Dunedin Study), reported similar findings [18]: DNAm age was determined by a 353, a 99, and a 71 CpGs epigenetic clock. While DNAm age estimated by the different epigenetic clocks correlated (r = 0.3–0.5), LTL and DNAm age estimates were not correlated (r = 0.02–0.05).

Breitling and colleagues studied an elderly German cohort of 1820 subjects (ESTHER cohort) [19]. DNAm age acceleration, determined by the difference of DNAm age (determined by Horvath’s 353 epigenetic clock) and the individual’s chronological age, did not correlate with telomere length in the studied cohort. Supporting evidence was provided by our group, who determined rLTL and DNAm age based on a seven CpGs epigenetic clock and IEAA in 1895 subjects of the BASE-II cohort, reporting a negligible association of rLTL with DNAm age (ß = −0.002, *p* = 0.011) and age acceleration (ß = −0.002, *p* = 0.007), suggesting that epigenetic clock and telomere length represent vastly different aspects of aging [16].

Chen and colleagues examined the relationship between LTL and DNAm age based on Hannum’s 71 CpGs epigenetic clock and the DNAm age acceleration (corrected for CD8^+^ T cells, memory CD8^+^ T cells and plasmablasts) in a total of 2539 subjects from three different cohorts (Women’s Health Initiative *n* = 804, the Framingham Heart Study *n* = 909, and the Bogalusa Heart study *n* = 826) [39]. While LTL and DNAm age estimate were reported to be independently associated with chronological age, LTL was weakly inversely correlated with the DNAm age acceleration in all three cohorts (r = −0.16 to −007) [39].

In conclusion, the absence of an association of rLTL with DNAm age and DNAm age acceleration observed in the LipidCardio cohort adds to the growing body of evidences indicating that DNAm age and DNAm age acceleration are biomarkers of ageing, independent of the mitotic age reflected by rLTL.

## 4. Materials and Methods

The LipidCardio study is a single-centre observational study, which was initiated to enable a comprehensive analysis of cardiovascular risk factors. A detailed description of the cohort can be found elsewhere [27]. Briefly, patients who underwent diagnostic cardiac catheterization at the Department of Cardiology (Charité-Universitätsmedizin Berlin, Campus Benjamin Franklin) in Berlin, Germany, between October 2016 and March 2018, were eligible for inclusion. Patients were recruited before undergoing the diagnostic procedure and were included after providing written informed consent, independent of their final diagnosis. Patients with troponin-positive acute coronary syndrome were not eligible.

The study was conducted in accordance with the Declaration of Helsinki and approved by the ethics committee of the Charité-Universitätsmedizin Berlin; approval number EA1/135/16, date of approval 27 June 2016.

### 4.1. Missing Data

For the purpose of this study, we chose to perform an available case analysis: Incomplete datasets were only excluded from the statistical data analysis if a missing affected one (or multiple) value of interest of the specific statistical test performed (pairwise deletion). Resulting deviations of the number of observations (*n*) for different statistical tests are indicated and detailed population statistics of all analyzed subpopulations are listed in the supplementary materials (Table S1).

### 4.2. Population Characteristics

To characterize the patient cohort phenotypically, participants’ data were collected inclusively, e.g., medical history, anthropometric measures, lifestyle factors (e.g., smoking, alcohol consumption, and physical activity), and a cardiovascular assessment with a coronary angiography. Blood was drawn for leukocyte genomic DNA isolation for rLTL and DNAm determination (routine laboratory testing and the biobank) during the cardiac catheterization, either from the radial or femoral artery sheath or via a peripheral intravenous access, after the administration of heparin.

### 4.3. DNA Extraction

Leukocyte genomic DNA was isolated from whole blood EDTA samples employing the “Plus XL manual kit” (LGC Genomics GmbH, Berlin, Germany) and according to the manufacturers protocol.

### 4.4. Determination of Relative Leukocyte Telomere Length

rLTL was determined according to Cowthon’s established protocol for telomere measurements by quantitative PCR and modified by Meyer et al. and Pfaffl’s mathematical model for relative quantification in RT-PCR [34,40,41]. 15 ng of genomic DNA were required for the protocol [34]. Amplified telomere, single copy gene (36B4), and reference sample (pooled from 10 randomly selected subjects) PCR products were measured in triplicates. Reference DNA with a concentration ranging from 0.37 to 30 ng was used, to generate the standard curve for each PCR plate and to determine PCR efficiency. PCR efficiency ranged from 1.57 to 1.65 (1.60 ± 0.02) for the telomere amplification and from 1.74 to 1.82 (1.77 ± 0.02) for the single copy gene amplification. For the individual efficiency of the qPCR was accounted by employing Pfaffl’s mathematical model for relative quantification [42]. The robustness of our measurements was indicated by a Pearson’s *R*^2^ = 0.99 for the telomeric standard curve and a *R*^2^ = 1.00 for the single copy standard curve (determined by regressing the Ct value onto the log of the DNA dilution).

Samples were excluded from further analysis, if the cycle threshold (Ct) value of the repeated measurements of telomeric or single copy gene PCR exceeded a coefficient of variance (CV) of 2% or of 5% in the pooled reference sample. Samples were excluded also if the mean rLTL of the individual sample differed from the cohorts mean rLTL by more than a factor one hundred.

Mean rLTL and standard deviations were calculated for the total cohort and tested for a correlation with chronological age. Moreover, mean rLTL in female and male subjects was compared.

### 4.5. DNA Methylation Assay

Epigenetic clocks are based on the weighted DNA methylation fraction of a varying choice and number of CpG sites selected by an elastic net (or lasso) penalized regression analysis.

For the purpose of this study we chose to adapt Vidal-Bralo’s and colleagues’ choice of CpGs and DNA methylation protocol with minor modifications, described and established by Vetter et. al. in our laboratory [14,16,28].

In summary: 500 ng of genomic leukocyte DNA sample underwent bisulfite conversion (EZ−96 DNA Methylation-Lightning Kit, ZYMO RESEARCH, Irvine, CA, USA) as described in the manufacturers’ protocol. A control sample of HPLC water was run alongside the samples on each multiplex plate during the entire procedure. Oligonucleotides for DNA amplification by multiplex polymerase chain reaction (mPCR) and multiplex single nucleotide primer extension (mSNuPE) were designed according to Vidal-Bralo et al. [14,28] (Table S3). The amplified DNA sample was cleaned prior to and following the mSNuPE. Differing from the protocol described by Vetter el al., 1.0 μL of template (instead of 0.1 μL) were added to 8.95 μL of HiDi formamide (Applied Biosystems, Waltham, MA, USA) and 0.05 μL GeneScan 120 LIZ size standard (Applied Biosystems, Waltham, MA, USA), to undergo analysis in a 3730 DNA Analyser (Applied Biosystems, Waltham, MA, USA). Only measurement spectra, meeting the pre-set quality requirements, were included in the data set for the stepwise penalize regression analysis to fit the model for DNAm age determination. Raw data were analyzed using GeneMapper software package 5 by Thermo Fisher Scientific (Waltham, MA, USA), and subsequent statistical analysis was performed using the SPSS Statistics 25 by IBM (Armonk, NY, USA) or Microsoft Excel 2016 (Redmond, WA, USA).

### 4.6. Determination of DNA Methylation (DNAm) Age

The formula for DNAm age determination was fitted by stepwise-penalized regression analysis, regressing the chronological age on the methylation fraction of the eight CpG sites previously described by Vidal-Bralo and colleagues [14,28]. DNAm fractions were determined by the peak height ratios, as suggested by Kaminsky et al. [42]. Chronological age was calculated by the difference of the inclusion date minus the date of birth, divided by 365.25 days (taking leap years into account). A Spearman’s correlation analysis was performed to test for a correlation of the DNA age and chronological age.

### 4.7. Determination of DNA Methylation (DNAm) Age Acceleration

In LipidCardio, DNA age acceleration was estimated by the residuals of the regression of the chronological age on the DNA methylation age, as suggested by Rosen et al. [43] and since the individual leukocyte distribution was unavailable. Samples were excluded from further statistical analysis, if the DNAm age acceleration differed more than three standard deviations of the mean of the study population.

To estimate the magnitude of the effect of age-associated changes of the blood cell distribution on the DNAm age acceleration, we retrieved data on chronological age, DNAm age, and intrinsic epigenetic DNAm age acceleration (IEAA) from a cohort of healthy subjects of the Berlin Aging Study II (BASE-II, *n* = 1395, age: 68.7 ± 3.7 years, 49.3% female). IAEE was defined as the residuals from regressing the chronological age on the DNAm age, adjusting for the individual’s neutrophils, monocytes, lymphocytes, and eosinophils count [16]. In both cohorts, BASE-II and LipidCardio, DNAm fractions were analyzed according to the same protocol. DNAm age was calculated based on a seven CpGs epigenetic clock, out of which six CpG sites were identical with the sites analysed in the current study. In addition to the IEAA, DNAm age acceleration was determined by the difference of DNAm age and chronological age, and the residuals from regressing the chronological age on the DNAm age, without adjusting for the individual’s blood cell count distribution. A bivariate correlation analysis was performed to explore the relationships of the three different methods for the determination of DNAm age acceleration. A *p*-value of 0.05 or lower was defined as statistically significant.

### 4.8. Exploring the Relationship Between Relative Leukocyte Telomere Length (rLTL) and Epigenetic Clock Variables

To explore the relationship between rLTL and the two epigenetic clock variables (DNAm age and DNAm age acceleration), a regression analysis was performed, separately regressing DNAm age and DNAm age acceleration on the rLTL, stepwise adjusting for chronological age, sex, and the lifestyle factors of alcohol consumption and smoking.

## Figures and Tables

**Table 1:** Characteristics of the LipidCardio participants for whom data on relative leukocyte telomere length (rLTL), DNA methylation (DNAm) age, and DNAm age acceleration were available (*n* = 773) (SD: standard deviation, *n*: number of observations, rLTL: relative leukocyte telomere length, BMI: body mass index, HDL-C: high density lipoprotein cholesterol, LDL-C: low density lipoprotein cholesterol, mg/dL: milligrams per deciliter).

Variables	Mean ± SD (*n*) or *n* and Percentage
Number of observations (*n*)	773
Female	244 (31.6%)
Chronological age (years)	69.68 ± 11.01
DNAm age (years)	69.67 ± 7.27
DNAm age acceleration/ residuals (years)	−0.01 ± 7.83
rLTL	0.79 ± 0.14
BMI	27.8 ± 4.8 (704)
Diabetes mellitus type II	208 (26.9%)
HDL- cholesterol (mg/dL)	51.23 ± 16.86 (739)
LDL- cholesterol (mg/dL)	99.28 ± 40.57 (741)
Hypertension	624 (80.7%)
Coronary heart disease	585 (75.8%) (772)
Myocardial infarction	234 (30.4%)
Ex-smoker/current smoker	470 (67.2%) (699)
Pack years	30.15 ± 28.9 (463)
Alcohol consumers	387 (56.0%) (691)
Alcohol consumed per week (units)	5.2 ± 6.2

**Table 2:** Model for the estimation of the DNA methylation age, resulting from the stepwise multiple linear penalized regression analysis with chronological age as the dependent variable (*n* = 785).

CpG Site	β	SE	*p*-Value
Intercept	101.629	2.741	5.03 × 10^−174^
cg19761273	−77.395	10.387	2.47 × 10^−13^
cg17471102	−27.062	4.711	1.33 × 10^−8^
cg02228185	−20.098	2.699	2.59 × 10^−13^
cg09809672	−18.923	4.178	7.00 × 10^−6^
cg10917602	−7.319	2.798	9.08 × 10^−3^
cg16386080	43.745	6.726	1.40 × 10^−10^
cg24768561	8.232	4.071	4.35 × 10^−2^
cg25809905 was excluded			

**Table 3:** Results of the linear regression of DNA methylation (DNAm) age and DNAm age acceleration on rLTL (*n* = 773).

	DNAm Age		DNAm Age Acceleration	
(Co-)Variables	β	*p*-Value	β	*p*-Value
Model 1: rLTL	4.69	0.14	1.69	0.59
Model 2: rLTL, Chronological age	3.30	0.14	3.07	0.17
Model 3: rLTL, Chronological age, Sex	3.00	0.18	2.74	0.22
Model 4: rLTL, Chronological age, Sex, Lifestyle factors (smoking, alcohol consumption)	3.00	0.18	2.76	0.22

## Supplementary Materials

Supplementary materials can be found at https://www.mdpi.com/1422-0067/20/12/3032/s1.

## Abbreviations

CpG	cytosine-phosphate-guanine
CpGs	cytosine-phosphate-guanine sites
DNA	Deoxyribonucleic acid
DNAm	DNA methylation
EEAA	cell-extrinsic epigenetic age acceleration
IEAA	cell-intrinsic epigenetic age acceleration
PCR	Polymerase chain reaction
rLTL	Relative leukocyte telomere length
SNuPE	Single nucleotide polymorphism extension
