# Supplementary material for: Exploring the Relationship of Relative Telomere Length and the Epigenetic Clock in the LipidCardio Cohort
Source: Int J Mol Sci. 2019 Jun 21;20(12):3032. doi: 10.3390/ijms20123032 (PMC6628615; doi:10.3390/ijms20123032)
Supplement: Supplementary file 1 [file ijms-20-03032-s001.pdf]

International Journal of
Molecular Sciences

MDPI

Supplementary Materials

Exploring the Relationship of Relative Telomere Length and the Epigenetic Clock in the LipidCardio Cohort

Verena L. Banszerus [1], Valentin M. Vetter [1], Bastian Salewsky [1], Maximilian König [2] and Ilja Demuth [1,3,*]

1 Charité–Universitätsmedizin Berlin, corporate member of Freie Universität Berlin, Humboldt-Universität zu Berlin and the Berlin Institute of Health, Lipid Clinic at the Interdisciplinary Metabolism Center, 13353 Berlin, Germany; verena-laura.banszerus@charite.de (V.L.B.); valentin.vetter@charite.de (V.M.V.); bastian.salewsky@gmx.de (B.S.)

2 Medizinische Klinik mit Schwerpunkt Nephrologie und Intensivmedizin, Charité-Universitätsmedizin Berlin; 13353 Berlin; Germany; koenig.maximilian@charite.de

3 Berlin-Brandenburg Center for Regenerative Medicine (BCRT), Charité Universitätsmedizin Berlin, 13353 Berlin; Germany

* Correspondence: ilja.demuth@charite.de; Tel.: +49-(0)30-450-569-143

1. Figures

Figure S1: LipidCardio subjects' chronological age versus relative leukocyte telomere length, indicating a lack of correlation between the above parameters (n = 948; R_s^2 = 3.59 × 10^{-4}, p = 0.56)

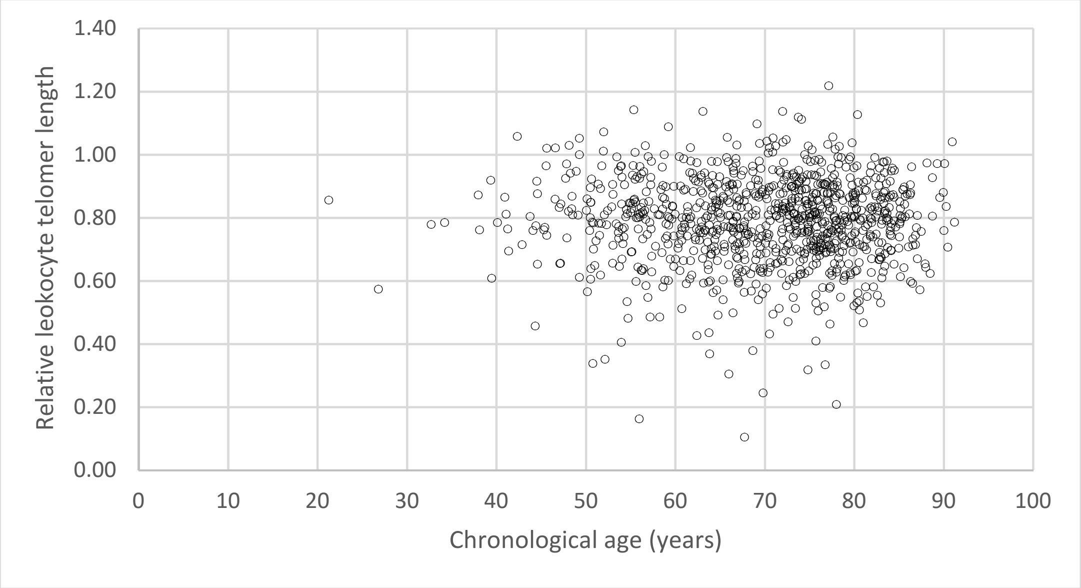

Figure S2: Linear relationship of the eight analysed cytosine-phosphate-guanine sites (CpGs) and the chronological age in years (n = 779) **a)** $R = -0.48$, $p = 5.32 \times 10^{-45}$ **b)** $R = 0.02$, $p = 0.68$ **c)** $R = 0.20$, $p = 3.27 \times 10^{-8}$ **d)** $R = 0.43$, $p = 1.28 \times 10^{-35}$ **e)** $R = -0.42$, $p = 6.05 \times 10^{-34}$ **f)** $R = 0.40$, $p = 4.03 \times 10^{-30}$ **g)** $R = -0.45$, $p = 3.88 \times 10^{-39}$ **h)** $R = -0.52$, $p = 3.08 \times 10^{-56}$ (n: Number of observations, R: Spearman´s-Rho, p: p-value)

a)

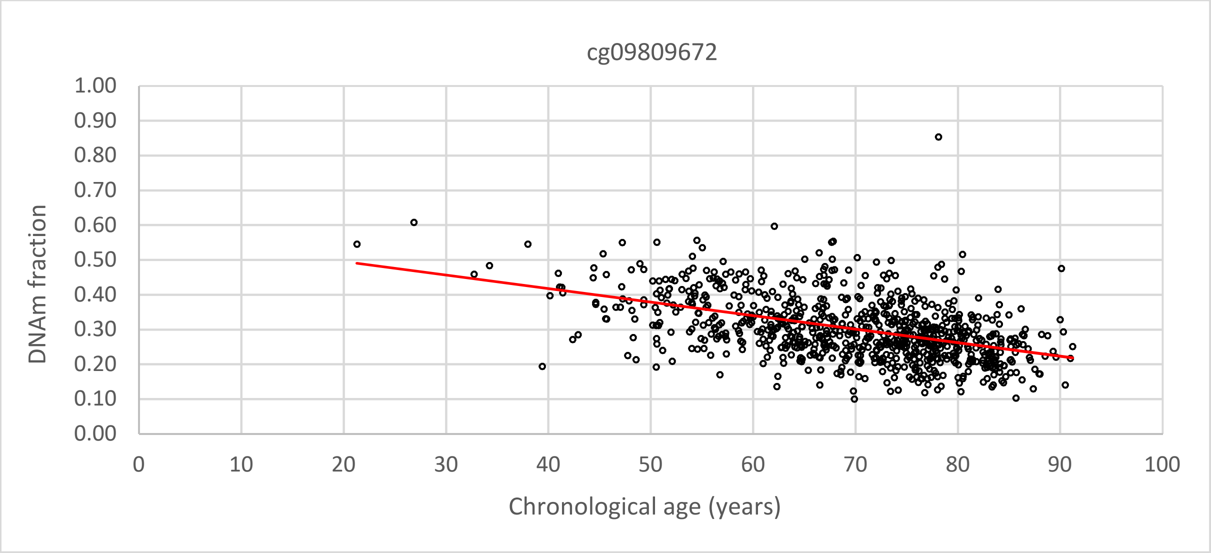

b)

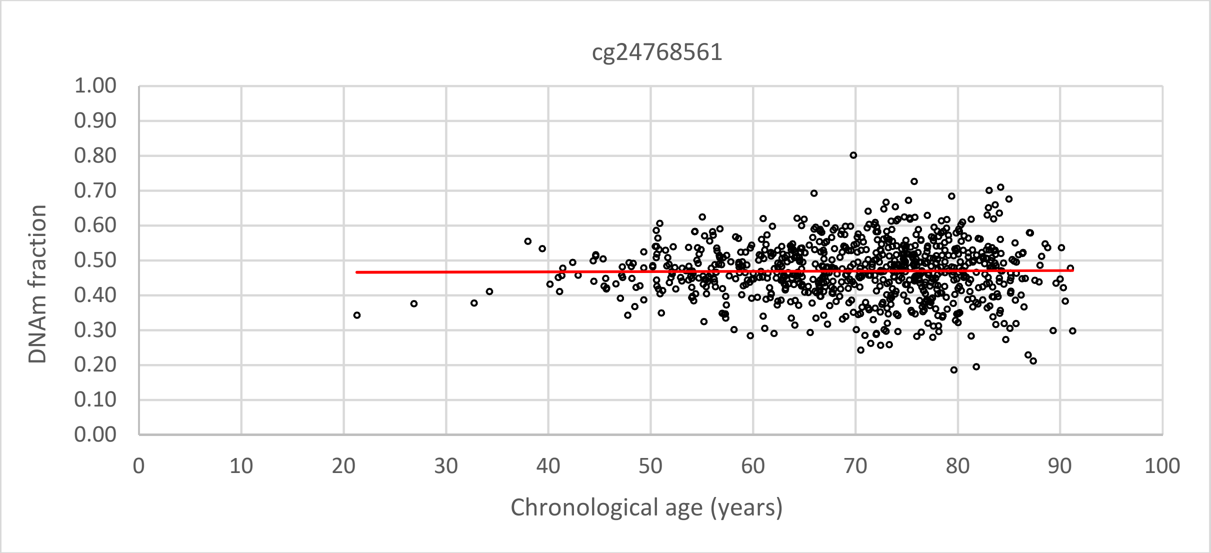

c)

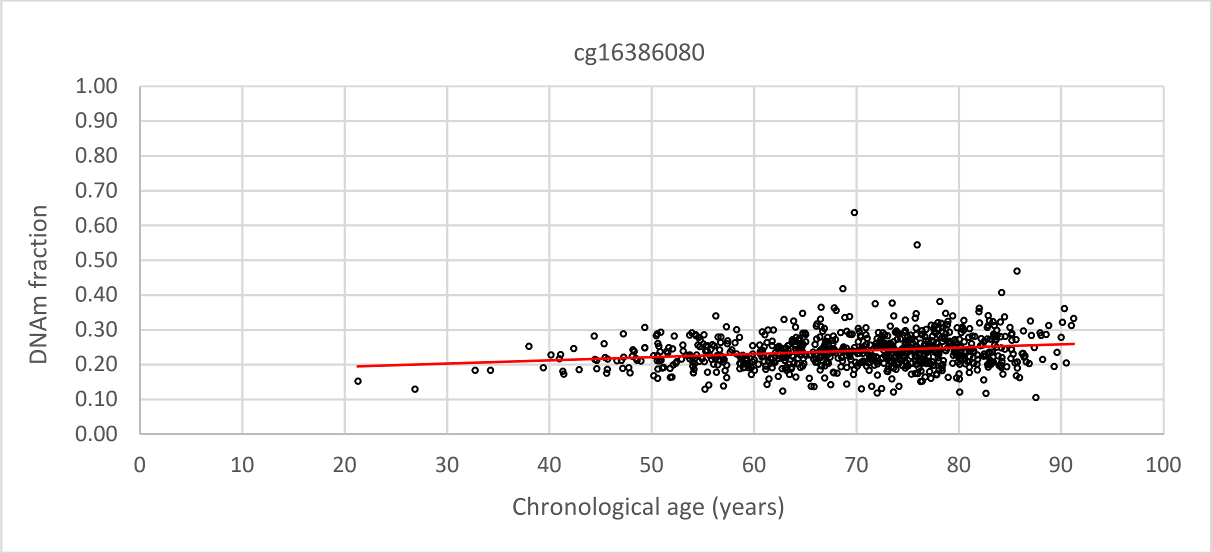
cg16386080
DNAm fraction
1.00
0.90
0.80
0.70
0.60
0.50
0.40
0.30
0.20
0.10
0.00
0
10
20
30
40
50
60
70
80
90
100
Chronological age (years)

d)

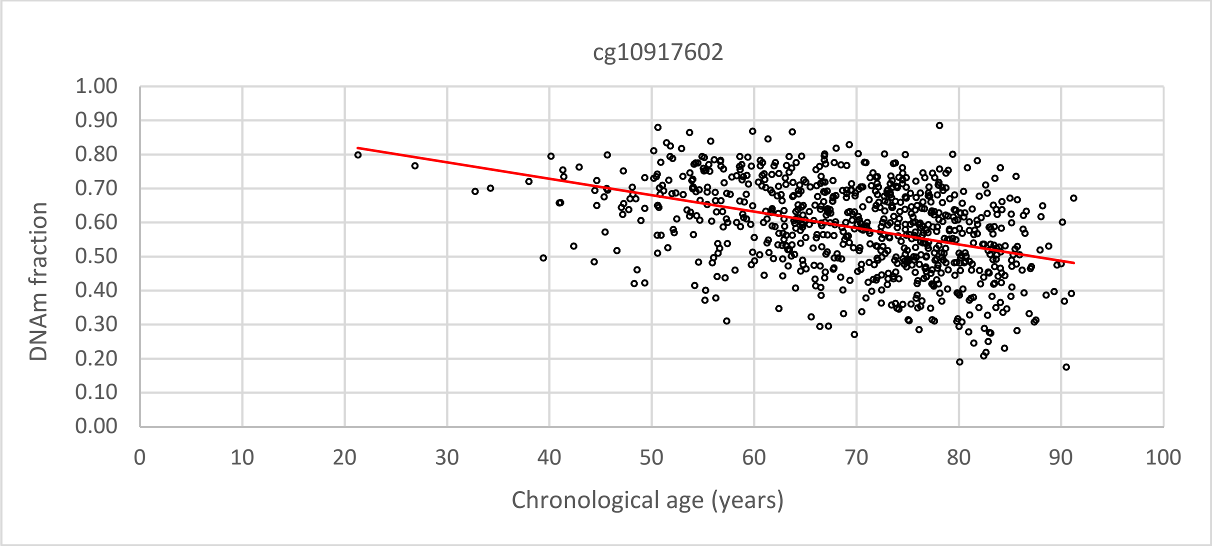
cg10917602
DNAm fraction
1.00
0.90
0.80
0.70
0.60
0.50
0.40
0.30
0.20
0.10
0.00
0
10
20
30
40
50
60
70
80
90
100
Chronological age (years)

e)

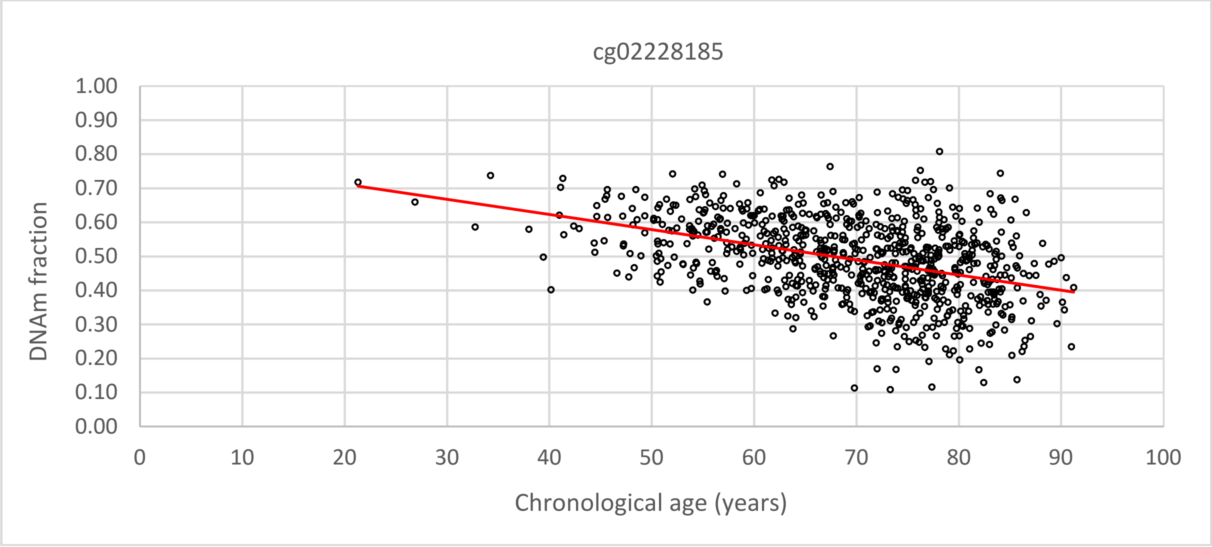
cg02228185
DNAm fraction
1.00
0.90
0.80
0.70
0.60
0.50
0.40
0.30
0.20
0.10
0.00
0
10
20
30
40
50
60
70
80
90
100
Chronological age (years)

f)

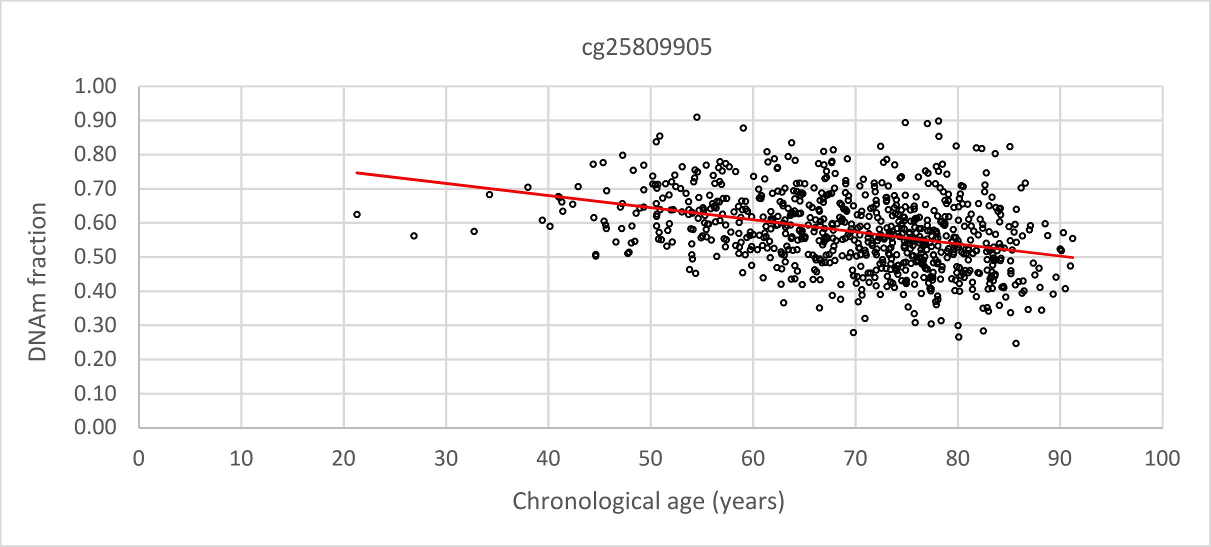
cg25809905
1.00
0.90
0.80
0.70
0.60
0.50
0.40
0.30
0.20
0.10
0.00
DNAm fraction
0
10
20
30
40
50
60
70
80
90
100
Chronological age (years)

g)

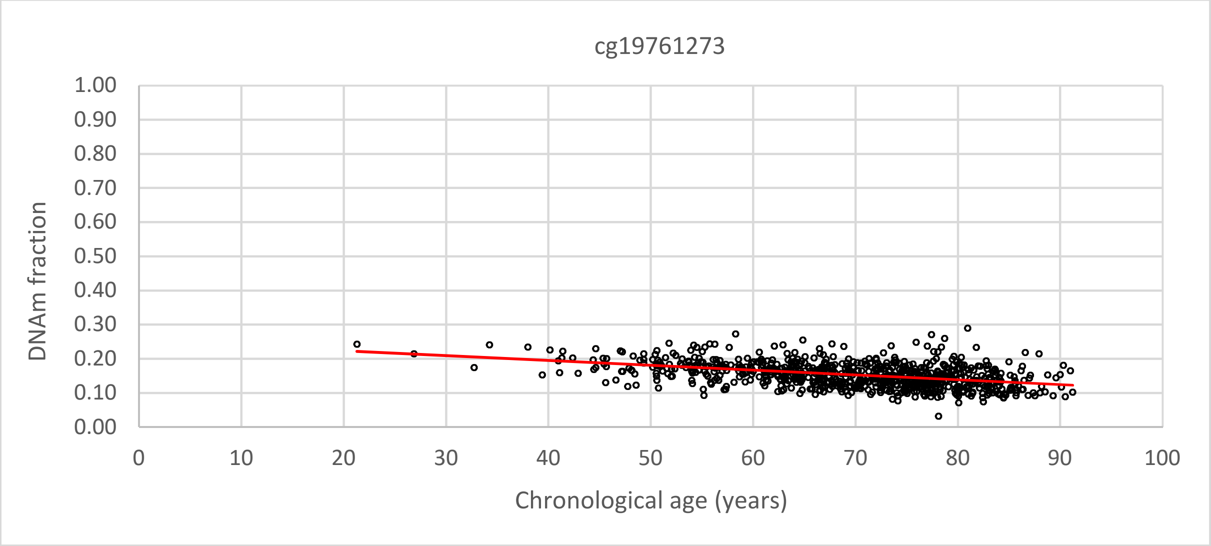
cg19761273
1.00
0.90
0.80
0.70
0.60
0.50
0.40
0.30
0.20
0.10
0.00
DNAm fraction
0
10
20
30
40
50
60
70
80
90
100
Chronological age (years)

h)

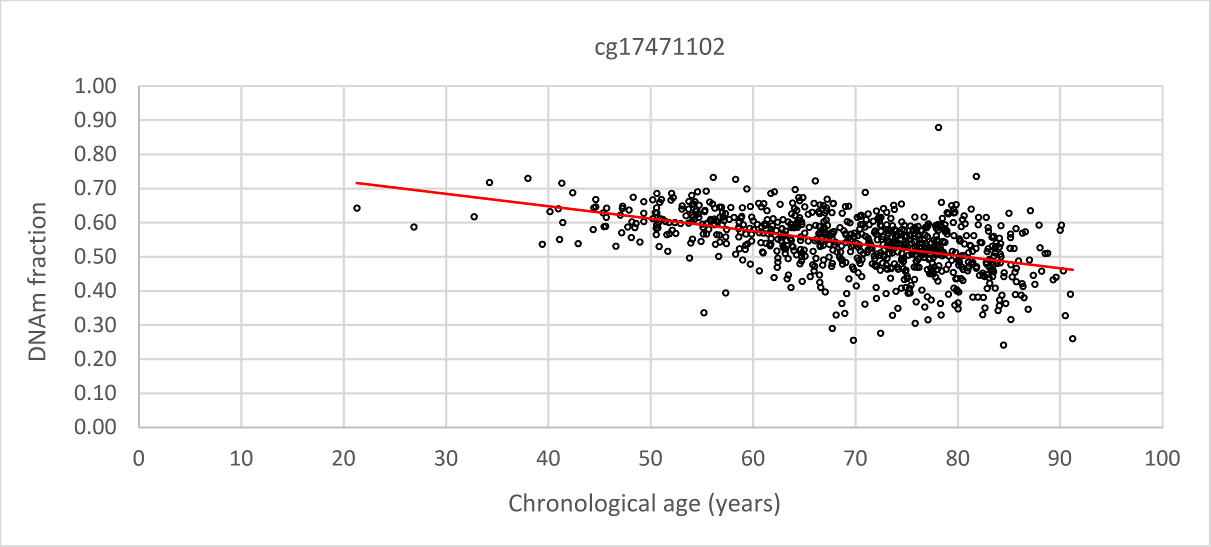
cg17471102
1.00
0.90
0.80
0.70
0.60
0.50
0.40
0.30
0.20
0.10
0.00
DNAm fraction
0
10
20
30
40
50
60
70
80
90
100
Chronological age (years)

Figure S3: Comparison of three alternative methods to determine DNAm age acceleration,. Data was retrieved from the Berlin Aging Study II ((BASE-II, *n* = 1395, age: 68.7 ± 3.7 years, 49.3% female), left: Intrinsic epigenetic age acceleration (IAEE) defined as the residuals from regressing the DNAm age on the chronological age, adjusting for the individuals neutrophils, monocytes, lymphocytes and eosinophils count, centre: the difference of the individual´s DNAm age (determined by an 7-CpGs epigenetic clock) and the chronological age, right: the residuals calculated from regressing the DNAm age (determined by an 7-CpGs epigenetic clock) on the chronological age.

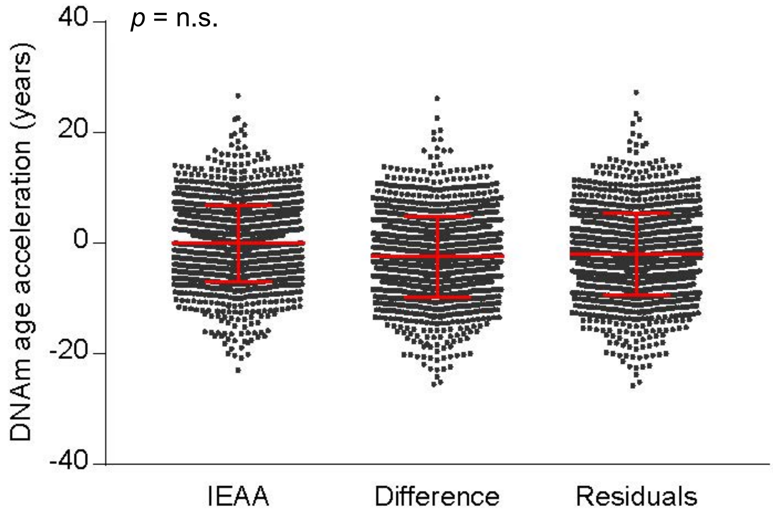

Figure S4: Relative telomere length (rLTL) versus **a)** DNAm age (*n*= 773) and **b)** DNAm age acceleration (*n*= 773)

a)

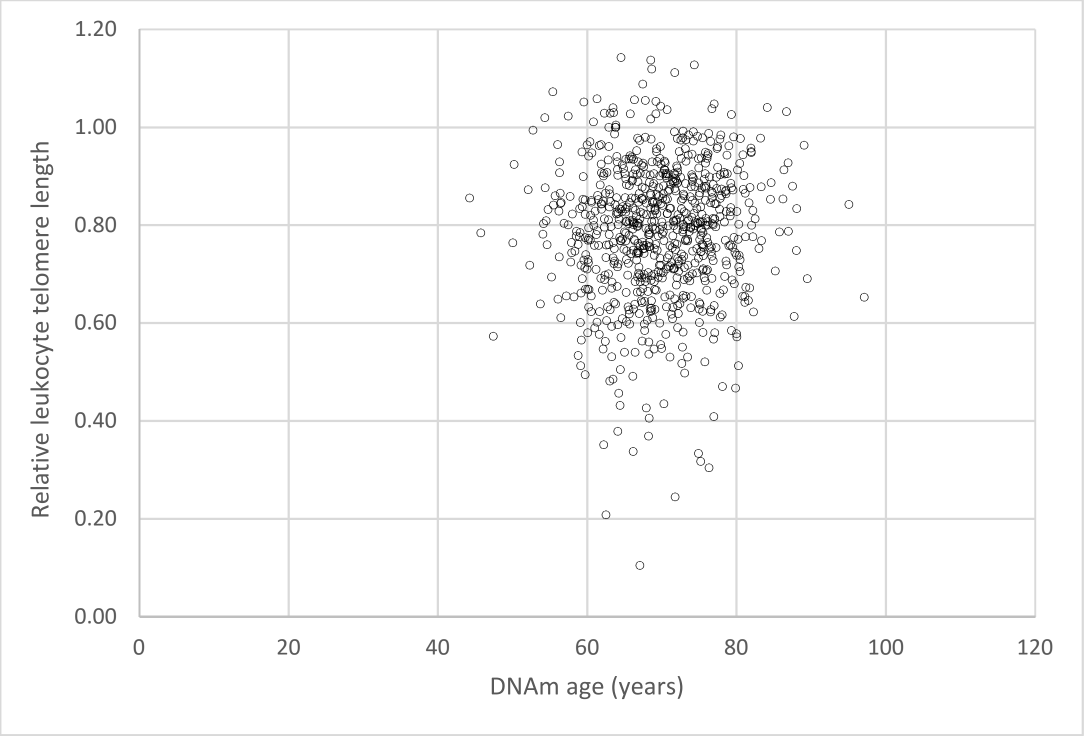

b)

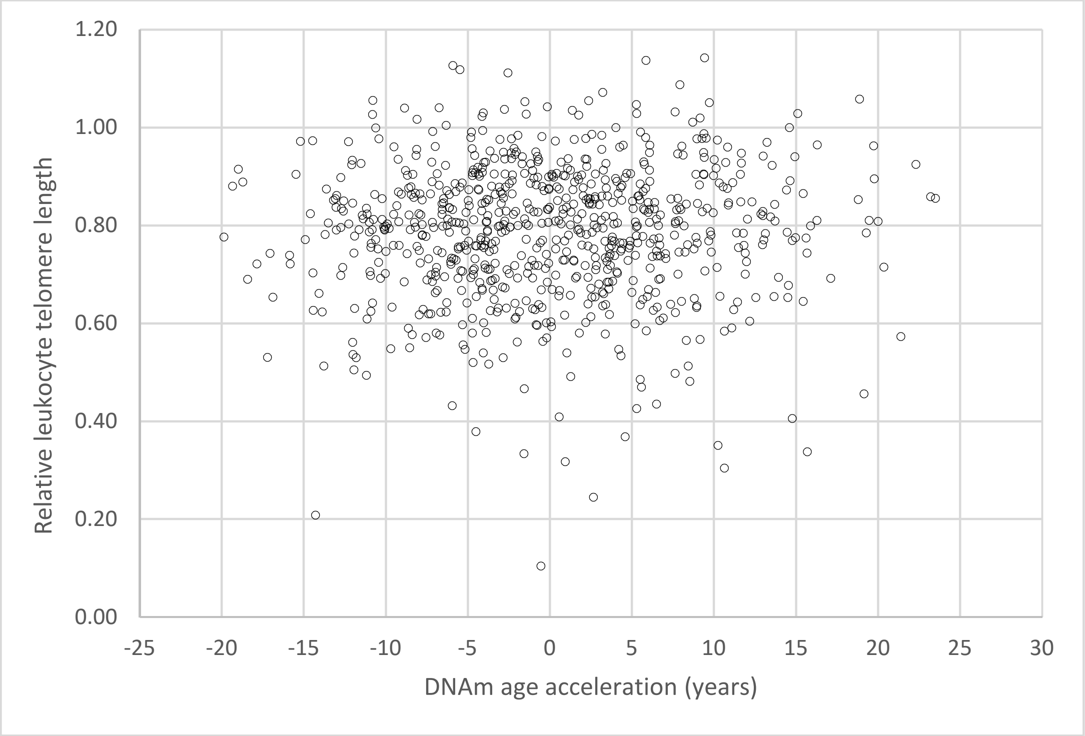

2. Tables

Table S1: Characteristics of the LipidCardio cohort, different populations of interests arise from the statistical approaches that deals with missing values by pairwise deletion, excluding incomplete datasets only if a missing affected one (or multiple) value of interest of the specific statistical test performed (SD: standard deviation, *n*: number of observations, rLTL: relative leukocyte telomere length, BMI: body mass index, HDL-C: high density lipoprotein cholesterol, LDL-C: low density lipoprotein cholesterol)

**Variables**	**Mean ± SD** (number of observations, if diverting from the total *n* stated above) or ***n*** **(percentage, total *n*)**				
**Population of interest**	**rLTL and DNAm Available**	**DNAm Age Available (After QC)**	**DNAm Age Available**	**rLTL Available**	**Total Cohort**
**Number of observations** (N)	773	779	785	948	1005
**Female**	244 (31.6%)	246 (31.6%)	248 (31.6%)	281 (29.6%)	303 (30.1%)
**Chronological age** (years)	69.68 ±11.01	71.80 ± 10.99	71.80 ± 11.11	71.86 ± 11.15	72.03 ± 11.07
**DNAm age** (years)	69.67 ± 7.27	69.43 ± 7.24	69.41 ± 7.43	69.41 ± 7.45 (779)	69.40 ± 7.43 (785)
**DNAm age acceleration/ residuals** (years)	−0.01 ± 7.83	−0.33 ± 8.81	−0.33 ± 8.24	−0.40 ± 8.26 (779)	0.00 ± 8.24 (785)
**rLTL**	0.79 ± 0.14	0.80± 0.14 (775)	0.80 ± 0.14 (781)	0.80 ± 0.14	0.80 ± 0.14 (950)
**BMI**	27.8 ± 4.8 (704)	27.8 ± 4.8 (709)	27.8± 4.8 (715)	27.8 ± 4.9 (874)	27.8 ± 4.9 (913)
**Diabetes mellitus type II**	208 (26.9%)	211 (27.1%)	213 (27.1%)	259 (27.3%)	270 (26.9%)
**HDL- cholesterol** (mg/dL)	51.23 ± 16.86 (739)	48.00 ± 16.83 (744)	48.00 ± 16.85 (750)	48.00 ± 16.60 (912)	48.00 ± 16.51 (964)
**LDL- cholesterol** (mg/dL)	99.28 ± 40.57 (741)	91.50 ± 40.57 (746)	91.50 ± 40.52 (752)	92.00 ± 40.45 (914)	92.00 ± 40.60 (961)
**Hypertension**	624 (80.7%)	628 (80.6%)	631 (80.4%)	769 (81.1%)	813 (80.9%)
**Coronary heart disease**	585 (75.8%) (772)	644 (82.8%) (778)	647 (82.4%) (785)	783 (82.6%)	829 (83.1%) (997)
**Myocardial infarction**	234 (30.4%)	237 (30.4%)	238 (30.3%)	292 (30.8%)	410 (40.8%)
**Smoking**					
Ex-smoker/ current smoker	470 (67.2%) (699)	473 (67.3%) (703)	475 (6.0%) (709)	583 (67.7%) (861)	604 (6.2%) (899)
Pack years	30.2 ± 28.9 (463)	24.0 ± 28.9 (466)	24.0± 28.8 (468)	24.0± 28.1 (575)	24.00 ± 27.9 (595)
**Alcohol consumption**					
Consumers	387 (56.0%)(691)	389 (49.9%)	392 (55.8%) (701)	471(55.5%) (849)	489 (55.4%) (883)
Units per week	3.0 ± 6.2 (380)	3.0± 6.2 (382)	3.0± 6.2 (385)	3.0 ± 6.4 (463)	3.0 ± 6.3 (479)

Table S2: Accuracy of DNAm age estimation with respect to chronological age across different age groups

**Age Group** (years)	**Number of Observations**	**Mean Chronological Age (SD)** (years)	**Mean DNAm Age Acceleration** (years)
< 60	154	52.64 (6.12)	8.96 (5.74)
60–70	195	65.34 (52.66)	2.45 (5.35)
70–80	286	75.10 (2.70)	−3.02 (5.59)
> 80	138	83.60 (2.70)	−7.26 (5.70)
21.28–91.22	773	69.68 (11.02)	−0.00 (7.83)

Table S3: Multiplex polymerase chain reaction (mPCR) and multiplex single nucleotide primer extension (mSnuPE) primer sequences (mSnuPE)

CpG Site	Forward mPCR Primer Sequence (Position)	Reverse mPCR Primer Sequence (Position)	mSNuPE Probe Sequence
cg09809672	TGAGAAATTTAGGAAGATAGTAAATG TTTA	AATTTATCCTCCCACCTACAAATTC C	TAACCAAACAACCAACIAACATCTTCTC
cg02228185	AATTATTTGGTGAAATGATTTTTTGTTA TA	AATAATTTTACCTCCAACCCTATTCT CTA	GGAGTATTTTTGGTTAAGTATTGGTTAGAGAATG G
cg19761273	GGAGGTTTTGATGTTTAGTTTGAAG	TCCACTCCTTATTTCCTTTACAAA	AACATTCAAATCCAACACAAATAAAAATATTAA CTCCICTCTCCAAACC
cg16386080	TTGGGGTAGGGGATTAAGTTAGTT	TCCCTTTTTACATCCAATACAATTTT	gccagcgtcagacatcatatgcagatacCCAATACAATTTTTAA AACCTACTCATATTCTAAACCTACTTTAAACC
cg17471102	GAAAGATTTTTGTTTGTGATTAGGG	AATTATCCCATTCTACCTTTTCCC	ATAAACCCTAATTCATAATATAACTAAACTAAC ACAAAATCCC
cg24768561	GTTTTGAGGTAAATGGGATTTT	CCCAACCAATAAACCAACAC	ATAACTAAAAACAAAAACTCAACCAATATCCTC AATCCAAAACCTTATAAAACC
cg25809905	GGGTTTTGTTTAGGGGAGTTTTT	TTTCCATCCAATCTTTCAACAATAC	attgatcgtggtgatatccgATAAATAATATACTCAATACT ATACCTACITATATTAACCCAC
cg10917602	TAGGAAGGTGGGAAGGGT	CATCCCCACCAAATTCTC	gatacCCCTCCAAACCAATCTAAACACCCTAAAAT AACIACTACAAATAAACAAAAAC

Table S4: Overview of studies examining the relationship of rLTL and DNAm age or DNAm age acceleration (N: Number of observations, TL: telomere length, qPCR: quantitative real-time polymerase chain reaction assay)

Cohort	n	Chronological Age (years)	Epigenetic Clock	Method of TL determination	Findings	Reference
**Lothian Birth Cohorts (LBC) of 1921 and 1936**	920 (LBC1936) and 414 (LBC1921)	70, 73 and 76 years (LBC1936) and 79, 87 and 90 years (LBC1921)	Hannum´s 71 CpGs epigenetic clock (and Horvath´s 353 CpGs epigenetic clock)	TL determined by qPCR	rLTL and DNAm age are independently associated with chronological age	Marioni R, Harris S, Shah S, McRae A, von Zglinicki T, Martin-Ruiz C, et al. The epigenetic clock and telomere length are independently associated with chronological age and mortality. *Int J Epidemiol*. 2016;45(2):424–32.
**Dunedin Study (one-year birth cohort)**	1037	26 and 38 years	Horvath´s 353 CpGs epigenetic clock, a 99 CpGs epigenetic clock and a Hannum´s 71 CpGs epigenetic clock	TL determined by qPCR	DNAm age estimated by the different epigenetic clocks correlated (r = 0.3–0.5); rLTL and DNAm age estimates were not correlated (r = 0.02–0.05)	Belsky D, Moffitt T, Cohen A, Corcoran D, Levine M, Prinz J, et al. Eleven Telomere, Epigenetic Clock, and Biomarker-Composite Quantifications of Biological Aging: Do They Measure the Same Thing? *Am J Epiderminology*. 2017;187(6):1220–1230.
**ESTHER cohort**	subsets of n = 969 and n = 851	62.1 ± 6.5 and 63.0 ± 6.7 years	Horvath´s 353 CpGs epigenetic clock	TL determined by qPCR	DNAm age acceleration, determined by the difference of methylation age and the individual´s chronological age, did not correlate with telomere length in the studied cohort.	Breitling L, Saum K-U, Perna L, Schöttker B, Holleczek B, Brenner H. Frailty is associated with the epigenetic clock but not with telomere length in a German cohort. Clin Epigenetics. *Clinical Epigenetics*; 2016;8(1):1–8, 21.
**Berlin Aging Study II (BASE-II)**	1,395 (older subset) and 424 (younger subset)	68.7 ± 3.7 years and 28.8 ± 3.5 years	a 7 CpGs epigentic clock adapted from Vidal-Bralo et al.	Relative TL determined by qPCR	negligible correlation of rLTL with DNAm age (ß = −0.002, p = 0.011) and IEAA (ß = −0.002, p = 0.007)	Vetter, V.; Meyer, A.; Karbasiyan, M.; Steinhagen-Thiessen, E.; Hopfenmüller, W.; Demuth, I. Epigenetic Clock and Relative Telomere Length Represent Largely Different Aspects of Aging in the Berlin Aging Study II (BASE-II). *J. Gerontol. Ser*. A 2018, 74, 27–32.

**Women's Health Initiative /Framing-ham Heart Study)/ Bogalusa Heart Study**	2539 (804/909/826)	ca. 50–80 (WHI) ca. 40–95 (FHS) ca. 25–50 (BHS)	Hannum´s 71 CpGs epigenetic clock, DNAm age acceleration (corrected for $CD8^+$ T cells, memory $CD8^+$ T cells and plasmablasts)	Leukocyte TL measured by the mean length terminal restriction fragments / Southern blot method	LTL and DNAm age independently associated with chronological age; LTL was weakly inversely correlated with the DNAm age acceleration ($r = -0.16$ to −007)	Chen B, Carty C, Kimura M, Kark J, Chen W, Li S, et al. Leukocyte telomere length , T cell composition and DNA methylation age. *Aging*. 2017;9(9):1983–1995.
**LipidCardio Study**	773	69.68 ± 11.01	a 7 CpGs epigentic clock adapted from Vidal-Bralo et al.	Relative TL determined by qPCR	No association of rLTL and DNAm age ($R = 0.045$), nor DNAm age acceleration ($R = 0.03$)	Banszerus V, Vetter V, Salewsky B, König M and Demuth I, Exploring the relationship of relative telomere length and the epigenetic clock in the LipidCardio cohort. *Int. J. Mol.* Sci. 2019 (this study)

© 2019 by the authors. Submitted for possible open access publication under the terms and conditions of the Creative Commons Attribution (CC BY) license (http://creativecommons.org/licenses/by/4.0/).
